# TGF‐β effects on adipogenesis of 3T3‐L1 cells differ in 2D and 3D cell culture conditions

**DOI:** 10.1002/2211-5463.13890

**Published:** 2024-10-08

**Authors:** Araya Umetsu, Megumi Watanabe, Tatsuya Sato, Megumi Higashide, Nami Nishikiori, Masato Furuhashi, Hiroshi Ohguro

**Affiliations:** ^1^ Department of Ophthalmology Sapporo Medical University School of Medicine Japan; ^2^ Department of Cardiovascular, Renal and Metabolic Medicine Sapporo Medical University School of Medicine Japan; ^3^ Department of Cellular Physiology and Signal Transduction Sapporo Medical University School of Medicine Japan

**Keywords:** 3D culture, 3T3‐L1 cell, adipogenesis, TGF‐β isoform

## Abstract

The TGF‐β superfamily plays a pivotal role in the regulation of adipogenesis, but little is known about the potential differential role of the three isoforms of TGF‐β, TGF‐β‐1~3. To further elucidate their role, two‐dimensionally (2D) and three‐dimensionally (3D) cultured 3T3‐L1 mouse preadipocytes were subjected to the following analyses: (a) qPCR analysis of adipogenesis‐related factors and major extracellular matrix protein (2D and /or 3D), (b) lipid staining by Oil Red O (2D) or BODIPY (3D), (c) Seahorse cellular metabolic measurement (2D), and (d) size and stiffness measurements of 3D 3T3‐L1 spheroids. In the 2D cultured 3T3‐L1 cells, mRNA expression levels of adipogenesis‐related genes and Oil Red O lipid staining intensity were significantly increased by adipogenesis and they were substantially decreased following treatment with 0.1 nm TGF‐β isoforms, with TGF‐β2 having the greater effects. Consistent with these results, treatment with TGF‐β2 resulted in suppression of mitochondrial and glycolytic functions in 2D cultured 3T3‐L1 cells. However, the inhibitory effect of TGF‐β on adipogenesis decreased under 3D spheroid culture conditions and TGF‐β isoforms did not affect adipogenesis‐induced (a) enlargement and downsizing of 3T3‐L1 spheroids, (b) increase in BODIPY lipid staining intensity, and (c) up‐regulation of the mRNA expression of adipogenesis‐related genes. The findings presented herein suggest that the three TGF‐β isoforms have different suppressive effects on adipogenesis‐related cellular properties of 2D cultured 3T3‐L1 cells and that their effects decrease under 3D spheroid culture conditions.

## Graphical abstract

This study investigates the impact of TGF‐β isoforms on adipogenesis using mouse preadipocytes in both 2D and 3D cultures. The results show that TGF‐β, especially TGF‐β2, significantly suppresses fat cell development and metabolism in 2D cultures, but these inhibitory effects are reduced in 3D spheroid cultures, highlighting the role of cellular environment in adipogenesis.

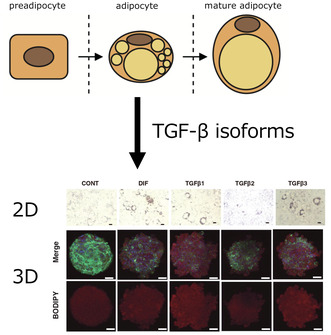

**Abbreviations**
2D: two‐dimensional
2‐DG: 2‐deoxyglucose
3D: three‐dimensional
ANOVA: analysis of variance
BMPs: bone morphogenetic proteins
C/EBP: CCAAT‐enhancer‐binding protein
CO_2_
: carbon dioxide
Col1: Collagen1
DEGs: differentially expressed genes
DIF: differentiation (meaning adipogenesis)
DMEM: Dulbecco's Modified Eagle Medium
ECAR: extracellular acidification rate
FABP4: fatty acid‐binding protein 4
FCCP: carbonyl cyanide p‐trifluoromethoxyphenylhydrazone
GDFs: growth and differentiation factors
GLUT4: glucose transporter 4
GO: Gene Ontology
IPA: ingenuity pathway analysis
mRNA: messenger ribonucleic acid
MSCs: mesenchymal stem cells
NT: non‐treatment
OCR: oxygen consumption rate
PFA: paraformaldehyde
PPARγ: peroxisome proliferator‐activated receptor γ
qPCR: quantitative Polymerase Chain Reaction
SMAD3: mothers against decapentaplegic homolog 3
TGF: transforming growth factor

It is well known that mesenchymal stem cells (MSCs) differentiate into multilineage cells including adipocytes and others [1, 2, 3, 4, 5, 6]. During the adipogenesis, MSCs migrate into an adipocyte lineage and are then initially converted into preadipocytes, an intermediate state of cells between MSCs and adipocytes, followed by differentiation into matured adipocytes [7, 8, 9, 10]. During these differentiation processes, various transcription factors including peroxisome proliferator‐activated receptor γ (PPARγ) and CCAAT‐enhancer‐binding protein (C/EBP) [11, 12, 13, 14] and various metabolic genes including fatty acid‐binding protein 4 (FABP4), glucose transporter 4 (GLUT4) and others are sequentially activated for adipocyte maturation as well as for maintaining these adipocyte phenotypes [10, 15]. As of this writing, although the molecular mechanism of differentiation of preadipocytes into adipocytes has been revealed in detail, little is known about the mechanism by which MSCs commit to preadipocytes. However, recent studies have suggested that the TGF‐β superfamily plays a pivotal role in the regulation of adipocyte commitment of MSCs.

It has been shown that TGF‐β inhibits the differentiation of osteoblasts [16] and myoblasts [17] in addition to the differentiation of adipocytes [18, 19]. In fact, *in vitro* experiments have shown that administration of TGF‐β blocks adipocyte differentiation [18, 20] and an *in vivo* study has shown that overexpression of TGF‐β in adipose tissue also inhibits adipocyte differentiation [21]. However, despite such TGF‐β‐induced suppression of adipocyte differentiation, high expression levels of endogenous TGF‐β were detected in adipose tissue *in vivo* [22] as well as in cultured preadipocytes and adipocytes [23, 24, 25].

The TGF‐β superfamily consists of various members including three TGF‐β isoforms (TGF‐β‐1, ‐2, and ‐3), Activin, Nodal, bone morphogenetic proteins (BMPs), growth and differentiation factors (GDFs), and other factors [26]. Despite the fact that the TGF‐β isoforms (TGF‐β‐1, ‐2, and ‐3) have approximately 70–80% homologous amino acid compositions [27] and the fact that they interact with the same receptors and signaling pathways, these isoforms exert diverse biological effects on physiological and pathological processes of wound healing [28, 29, 30, 31] as well as the pathogenesis of glaucoma [32, 33, 34, 35]. These findings collectively suggest that the three TGF‐β isoforms (TGF‐β‐1, ‐2, and ‐3) may also have different effects on adipogenesis. However, as far as we surveyed, there have been limited studies related to this issue.

Therefore, in this study, to elucidate the effects of TGF‐β isoforms (TGF‐β‐1, ‐2, and ‐3) on adipogenesis, lipid production and gene expression of adipogenesis‐related molecules of 2D and 3D cultures of 3T3‐L1 cells were evaluated.

## Materials and methods

### Adipocyte cultures and the differentiation of 3T3‐L1 cells

3T3‐L1 preadipocytes (#JCRB9014, Japanese Collection of Research Bioresources Cell Bank, Ibaraki City, Japan) were grown in 2D cultures and subjected to further 3D cultures using a hanging droplet culture plate (# HDP1385, Sigma‐Aldrich, Inc., St. Louis, MO, USA) for 7 days as described previously [36, 37, 38]. The 7‐day culture period was decided on the basis of results of our previous study showing that 3D 3T3‐L1 spheroid maturation is complete and that the cultures are stabilized after a 7‐day culture period [39]. Therefore, fresh 3D 3T3‐L1 spheroids were used in the experiments as in our previous studies [36, 37, 39]. Adipogenesis in the 2D or 3D cultured 3T3‐L1 cells [36, 37, 38] was initiated by the addition of 250 nm dexamethasone and 10 nm T3 for 2 days and with 10 μm troglitazone and 1 μg·mL^−1^ insulin for the next 3 days. To study the effects of TGF‐β isoforms on adipogenesis of 3T3‐L1 cells, 0.1 or 1 ng·mL^−1^ TGF‐β1 (#209‐21571, Fujifilm, Tokyo, Japan), TGF‐β2 (#200‐19911, Fujifilm) or TGF‐β3 (#203‐21591, Fujifilm) was administered during the 7‐day cultures with concentration ranges of TGF‐β isoforms based on previous studies [18, 40, 41].

### Lipid staining by oil red O (2D) or BODIPY (3D)

For investigation of lipid production during adipogenesis, 4% paraformaldehyde (PFA)‐fixed 2D and 3D cultured 3T3‐L1 cells were subjected to Oil Red O staining and BODIPY lipid staining, respectively, and confocal images were obtained basically as shown in our previous reports [36, 37, 38].

### Measurement of real‐time mitochondrial and glycolytic functions

The oxygen consumption rate (OCR) and extracellular acidification rate (ECAR) of 2D cultured 3T3‐L1 cells were measured for assessment of the mitochondrial and glycolytic functions of the cells, respectively, using a seahorse XFe96 Bioanalyzer (Agilent Technologies, Santa Clara, CA, USA) as basically described in our previous reports [42, 43, 44]. Briefly, 20 × 10^3^ 2D cultured 3T3‐L1 preadipocytes (DIF^−^) or their adipogenesis that were not treated (non‐treated control, NT) or treated with solutions of 0.1 nm TGF‐β‐1, ‐2 or ‐3 as described above were seeded into wells of a 96‐well Seahorse measurement analytical plate before the day of the assay. On the day of the assay, after exchanging the culture medium with Seahorse XF DMEM assay medium (pH 7.4, Agilent Technologies, #103575‐100) supplemented with 5.5 mm glucose, 2.0 mm glutamine, and 1.0 mm sodium pyruvate, the plate was incubated in a CO_2_‐free incubator at 37 °C for 1 h before the assay. Then the basal OCR and ECAR values in the cells were determined by using the Seahorse XFe96 Bioanalyzer and the samples were further analyzed after supplementation with 2.0 μm oligomycin, 5.0 μm carbonyl cyanide p‐trifluoromethoxyphenylhydrazone (FCCP), 1.0 μm rotenone and antimycin A, and 10 mm 2‐deoxyglucose (2‐DG). After completion of the assay, the assay buffer was removed and 10 μL CelLytic™ MT Cell Lysis Reagent (Sigma‐Aldrich, Inc.) was added to each well to measure the protein concentration for correction of OCR and ECAR values.

### Measurement of the mechanical properties including size and solidity of 3D 3T3‐L1 spheroids

The mechanical properties including size and solidity of the 3T3‐L1 3D spheroids obtained as described above were evaluated basically reported as reported previously [45, 46]. In brief, the mean sizes (μm^2^) of the 3D spheroids were determined as the largest cross‐sectional area using phase contrast microscopy images. For the solidity measurement, a single living 3D spheroid was compressed to its semi‐diameter during a period of 20 s using a micro spheroid compressor system (MicroSquisher, CellScale, Waterloo, ON, Canada). The required force (μN) and semi‐diameter (μm) were simultaneously measured by a micro‐pressure sensor and micro‐monitoring camera, respectively, equipped in the system. To estimate the mechanical solidity of the 3D spheroid, the index of the required force (μN) per semi‐diameter (μm) was used.

### Quantitative PCR

Total RNA extraction (RNeasy mini kit, Qiagen, Valencia, CA, USA), reverse transcription (SuperScript IV kit, Invitrogen, Carlsbad, CA, USA), and real‐time PCR (Applied Biosystems/Thermo Fisher Scientific, Waltham, MA, USA) were performed as described previously [36, 37, 38]. Normalization of the cDNA levels of the respective genes was performed by the standard curve method for relative quantitation using the 36B4 (*Rplp0*) gene as a housekeeping gene [47]. Sequences of the primers and Taqman probes used in this study are shown in Table S1.

### Statistical analysis

All statistical analyses were performed using graphpad prism 8 (GraphPad Software, San Diego, CA, USA). To analyze the difference between groups, a grouped analysis with two‐way analysis of variance (ANOVA) followed by Tukey's multiple comparison test was performed. Data are presented as arithmetic means ± standard error of the mean (SEM).

## Results

### Effects of TGF‐β isoforms on adipogenesis of 2D cultured 3T3‐L1 cells

**Fig. 1:**
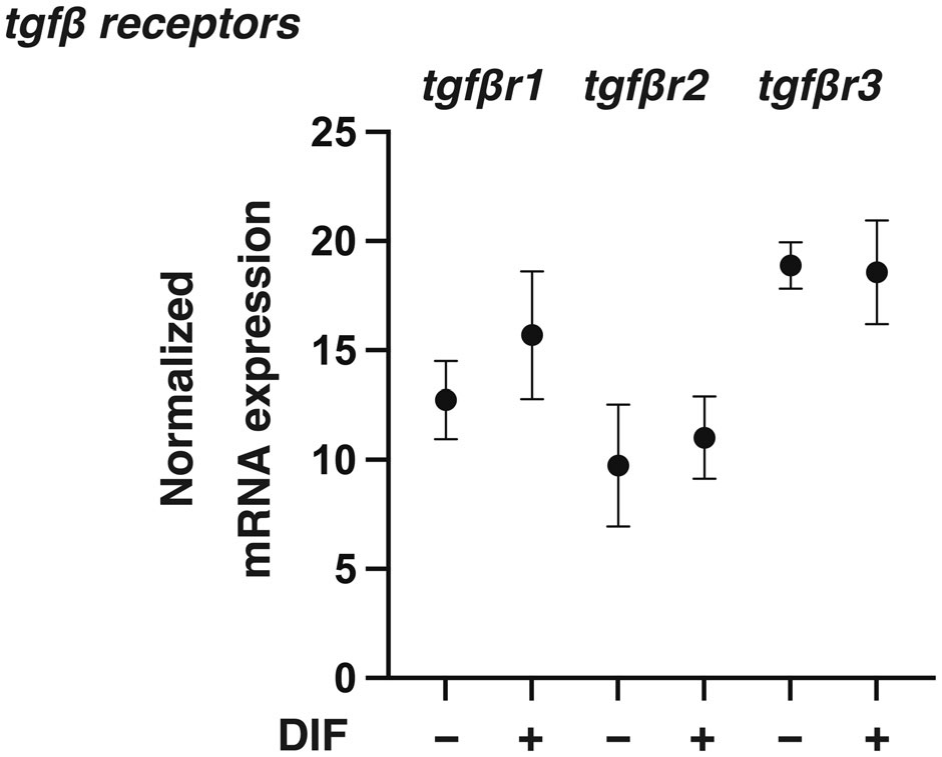
mRNA expression of TGF‐β isoform receptors (TGFβ1R, TGFβ2R, and TGFβ3R) of 2D cultured 3T3‐L1 cells before and after adipogenesis. 2D cultures of 3T3‐L1 preadipocytes (DIF^−^) or their adipogenesis (DIF^+^) were subjected to qPCR analysis of TGF‐β isoform receptors (*tgfbr1*, *tgfbr2*, and *tgfbr3*). All of the experiments were performed in triplicate (*n* = 3) using fresh preparations, each of which consisted of five specimens. The analysis is done by ANOVA with SD shown as error bars.

**Fig. 2:**
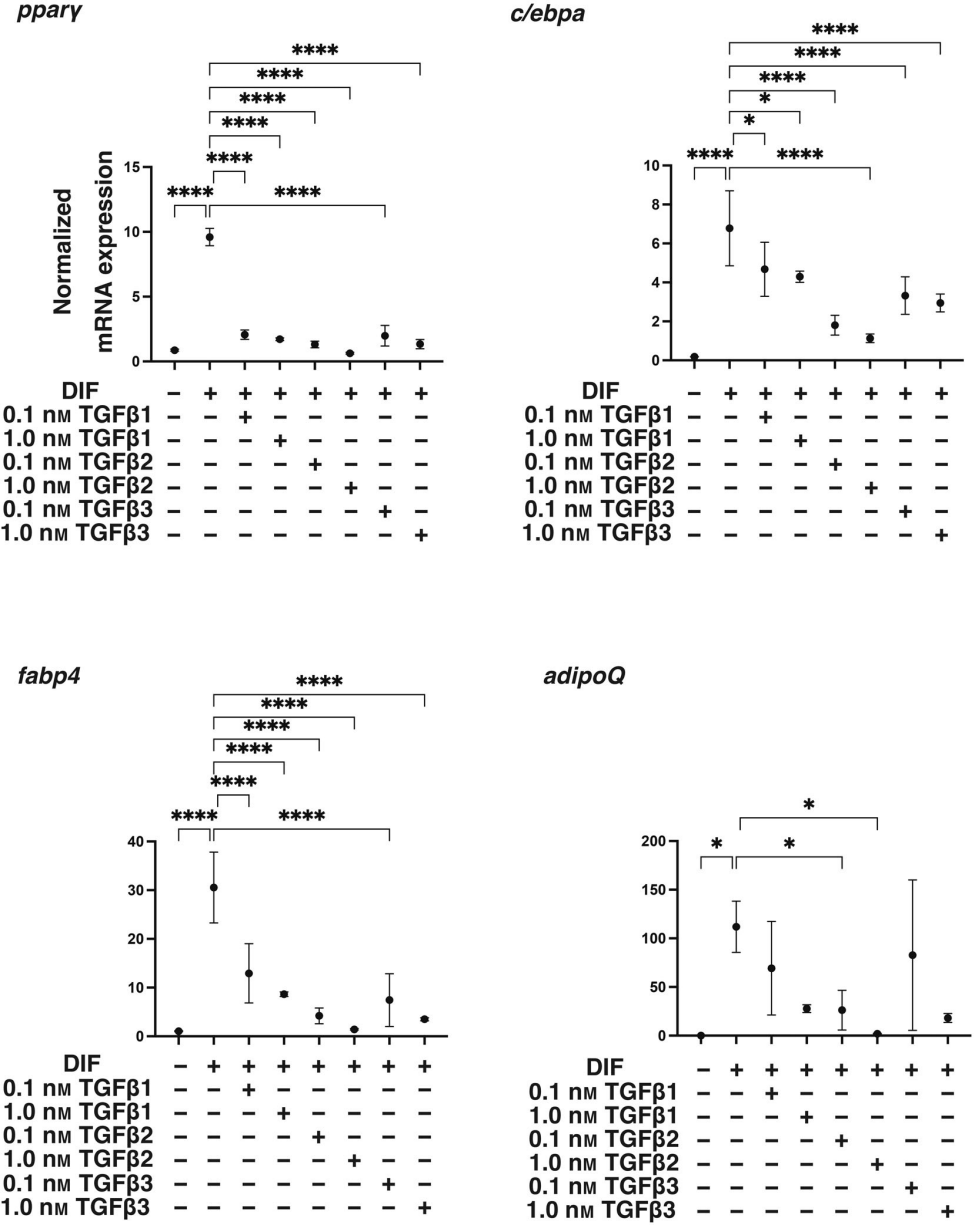
Effects of TGF‐β isoforms on mRNA expression of adipogenesis‐related genes of 2D cultured 3T3‐L1 cells during their adipogenesis. 2D cultures of 3T3‐L1 preadipocytes (DIF^−^) or their adipogenesis (DIF^+^) with or without treatment with 0.1 or 1 nm TGF‐β1, TGF‐β2 or TGF‐β3 were subjected to qPCR analysis of adipogenesis‐related genes including *pparγ*, *c/ebpa, adipo q*, and *fabp4*. All of the experiments were performed in triplicate (*n* = 3) using fresh preparations, each of which consisted of five specimens. ****P < 0.001. The analysis is done by ANOVA with SD shown as error bars.

**Fig. 3:**
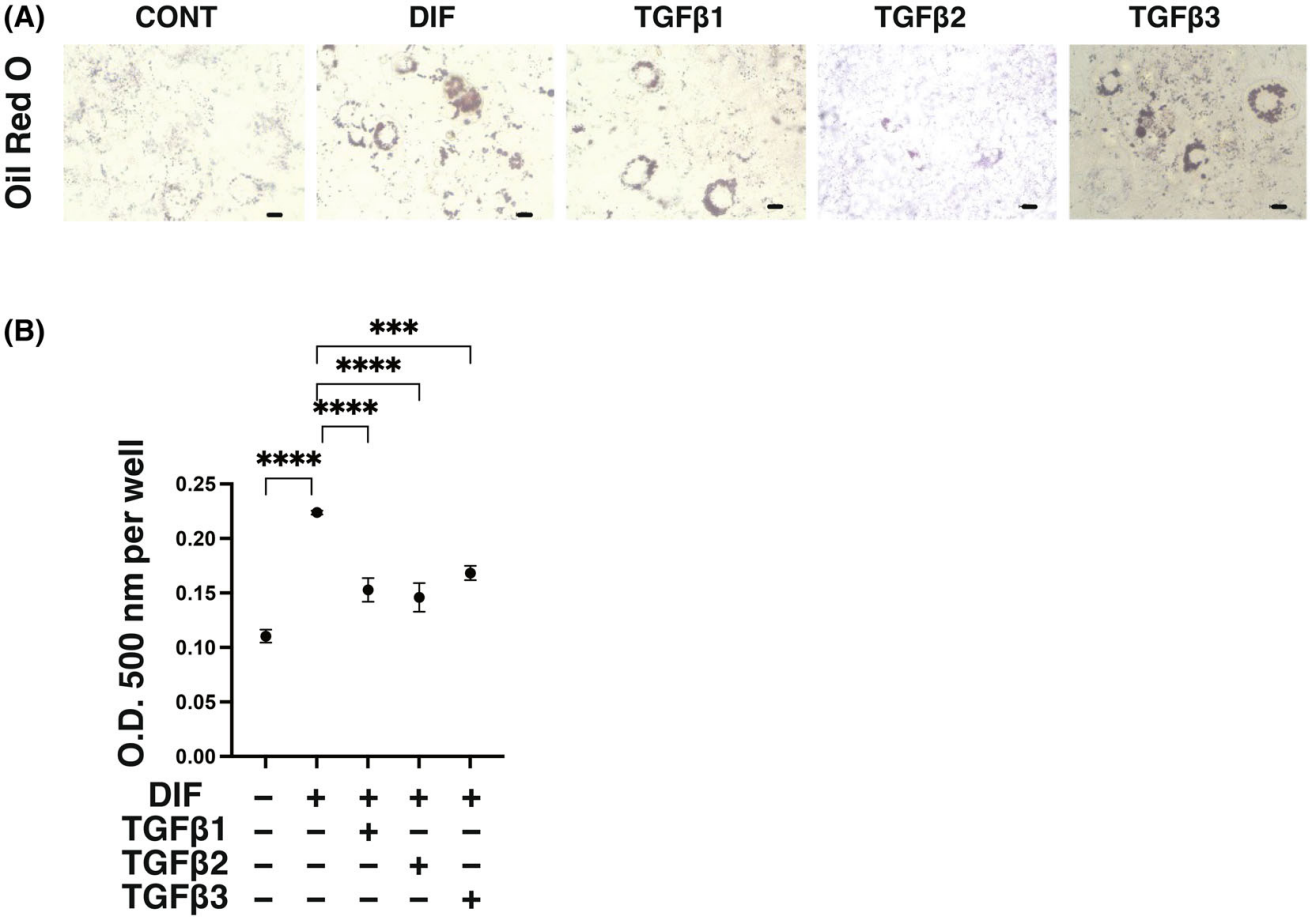
Effects of TGF‐β isoforms on lipid production of 2D cultured 3T3‐L1 cells during their adipogenesis. 2D cultures of 3T3‐L1 preadipocytes (DIF^−^) or their adipogenesis (DIF^+^) with or without treatment with 0.1 nm TGF‐β1, TGF‐β2 or TGF‐β3 were subjected to analysis by Oil Red O lipid staining. Representative phase contrast images (each scale bar: 100 μm, panel A) and plots of staining intensities (O.D., panel B) are shown. All of the experiments were performed in triplicate (*n* = 3) using fresh preparations, each of which consisted of five specimens. ****P < 0.001. The analysis is done by ANOVA with SD shown as error bars.

**Fig. 4:**
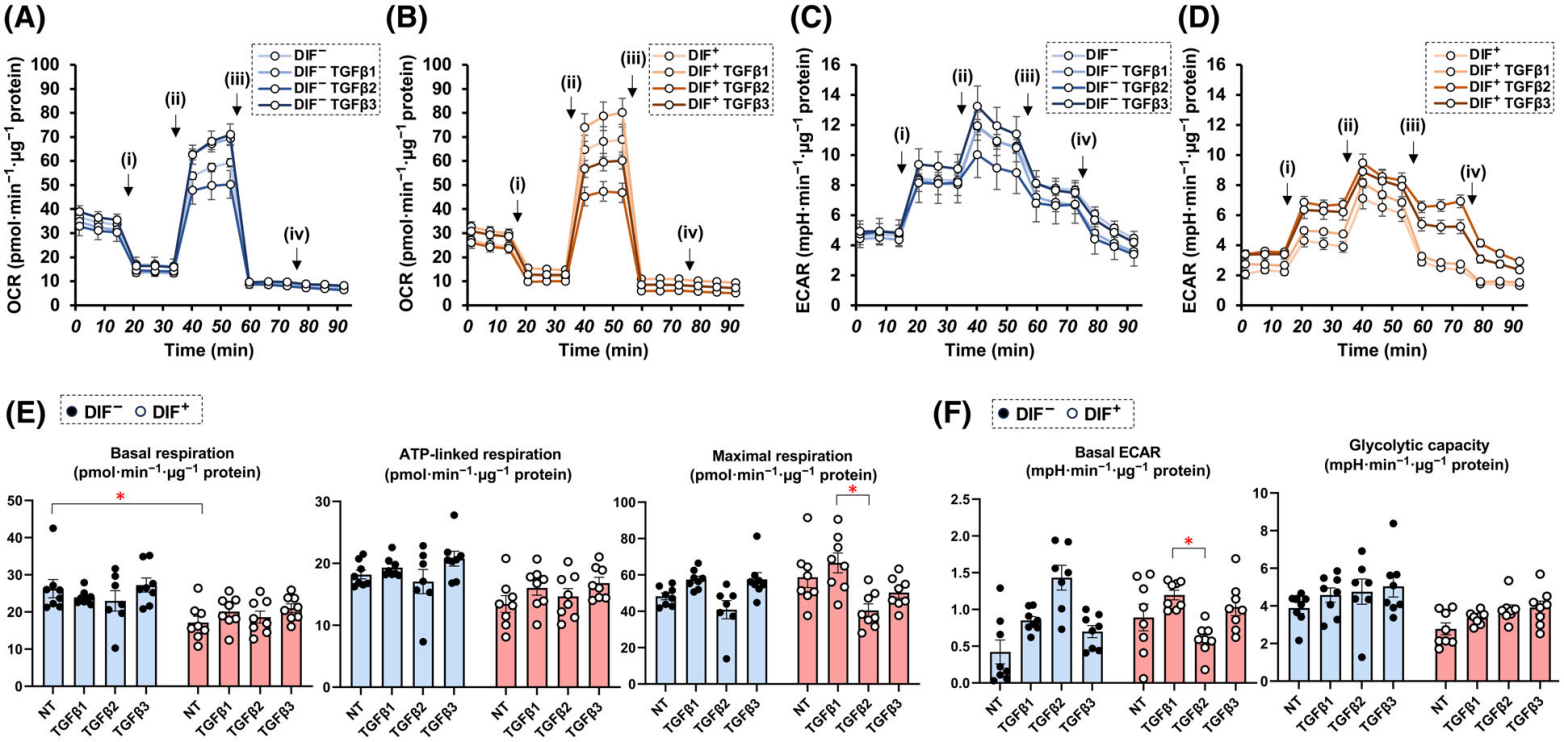
Effects of TGF‐β isoforms on mitochondrial and glycolytic functions in 2D cultured 3T3‐L1 cells. 2D cultures of 3T3‐L1 cells were prepared under several sets of conditions: Firstly, preadipocytes of 3T3‐L1 cells without (NT) or with adipogenesis (DIF^+^), or with 0.1 nm TGF‐β1, TGF‐β2 or TGF‐β3. These specimens were then analyzed by real‐time metabolic function analysis. OCR (panel A: preadipocyte, panel B: with the induction of adipogenesis) and ECAR (panel C: preadipocyte, panel D: with the induction of adipogenesis) were calculated at baseline and then they were further measured by subsequent supplementation with oligomycin (i), FCCP (ii), rotenone/antimycin A (iii), and 2‐Deoxy‐D‐glucose (iv). Mitochondrial function (panel E) and glycolytic function (panel F) were shown as follows: Basal respiration: subtracting OCR with rotenone/antimycin A from OCR at baseline, ATP‐linked respiration: the difference in OCR after the addition of oligomycin, Maximal respiration: subtracting the OCR with rotenone/antimycin A from OCR with FCCP, Basal ECAR: subtracting ECAR with 2‐DG from ECAR at baseline, Glycolytic capacity: subtracting ECAR with 2‐DG from ECAR with oligomycin. *P < 0.05. The analysis is done by ANOVA with SD shown as error bars.

To elucidate the biological roles of TGF‐β isoforms on adipogenic differentiation (DIF^+^), adipogenesis was induced in 2D and 3D cultured 3T3‐L1 preadipocytes in the absence or presence of different concentrations (0.1 or 1 nm) of TGF‐β‐1~‐3 for analysis of the mRNA expression of adipogenesis‐related genes including *Pparγ*, *c/ebpa, adipo q*, and *fabp4*. In advance, we confirmed presence of mRNA expressions of receptors for *TGF‐β1 (tgfβr1)*, *TGF‐β2 (tgfβr2)* and *TGF‐β3 (tgfβr3)* in 3T3‐L1 cells and that those levels were comparable before and after adipogenesis (DIF^+^) (Fig. 1). As shown in Fig. 2, the expression of all of the adipogenesis‐related genes was remarkably up‐regulated upon adipogenesis (DIF^+^) as observed in our previous studies [36, 37, 48, 49], and the changes in expression were substantially inhibited by TGF‐β isoforms. However, the degrees of suppression of the adipogenesis (DIF^+^)‐induced up‐regulation of those four genes were different depending on the isoforms of TGF‐β and their concentrations. That is, the effects of TGF‐β‐2 were more pronounced than those of TGF‐β‐1 and TGF‐β‐3, and the effects were in a concentration‐dependent manner for TGF‐β‐2 but not for TGF‐β‐1 or TGF‐β‐3 at the concentrations used. Based on these results, the following studies including lipid staining by Oil Red O, and analysis of cellular metabolic function by a seahorse bioanalyzer were performed using TGF‐β isoforms at a fixed concentration of 0.1 nm. In an analysis by Oil Red O lipid staining, diverse effects of the three TGF‐β were also observed (Fig. 3). Namely, (a) upon adipogenesis (DIF^+^), (a) the staining intensities were significantly increased, (b) lipid synthesis was suppressed by all three TGF‐β isoforms, and (c) the suppressive effects were potent in the order of TGF‐β‐2, TGF‐β‐1 and TGF‐β‐3, the order being almost the same as that for mRNA expression levels of the adipogenesis‐related genes as shown in Fig. 2. In addition, oxygen consumption rate (OCR) and extracellular acidification rate (ECAR) as indices for cellular mitochondrial and glycolytic functions, respectively, were also different depending on the TGF‐β isoforms, that is, both indices were decreased by TGF‐β2 treatment compared to those in the case of treatment with other TGF‐β isoforms in 2D cultured 3T3‐L1 cells with the induction of adipogenesis (DIF^+^) (Fig. 4). These results suggest that TGF‐β2 has an exclusively inhibitory effect on adipogenesis in 2D cultured 3T3‐L1 cells compared to the other TGF‐β isoforms.

### Effects of TGF‐β isoforms on adipogenesis of 3D 3T3‐L1 spheroids

**Fig. 5:**
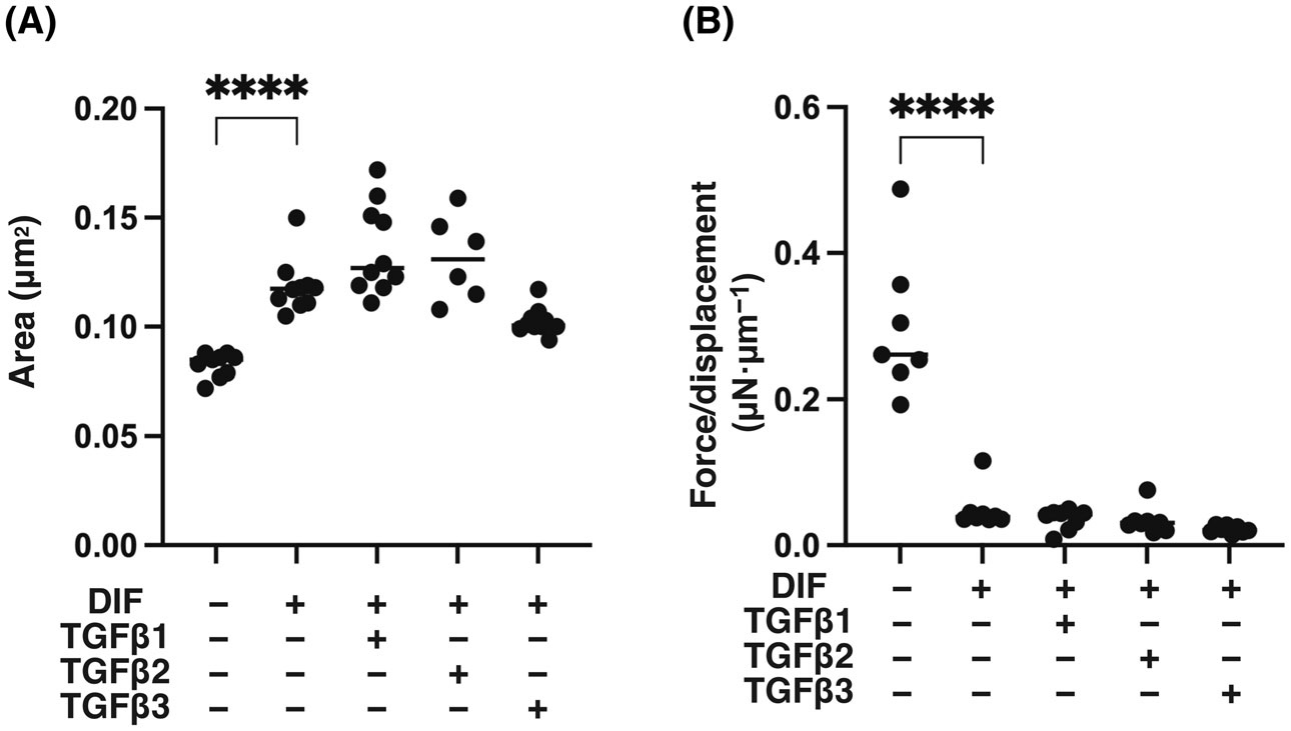
Effects of TGF‐β isoforms on mechanical properties including sizes and stiffness of 3D 3T3‐L1 spheroids during their adipogenesis. 3D spheroids of 3T3‐L1 preadipocytes without adipogenesis (DIF^−^) or their adipogenesis (DIF^+^) with or without treatment with 0.1 nm TGF‐β1, TGF‐β2 or TGF‐β3 (*n* = 16 spheroids each) were prepared. Their mean sizes measured are shown in panel (A). By using a micro‐squeezer, and the requiring force (μN) to compress a single spheroid into its semi‐diameter (μm) during a period of 20 s, mechanical solidity was measured was shown in panel (B). *P < 0.05, ****P < 0.001. The analysis is done by ANOVA with SD shown as error bars.

**Fig. 6:**
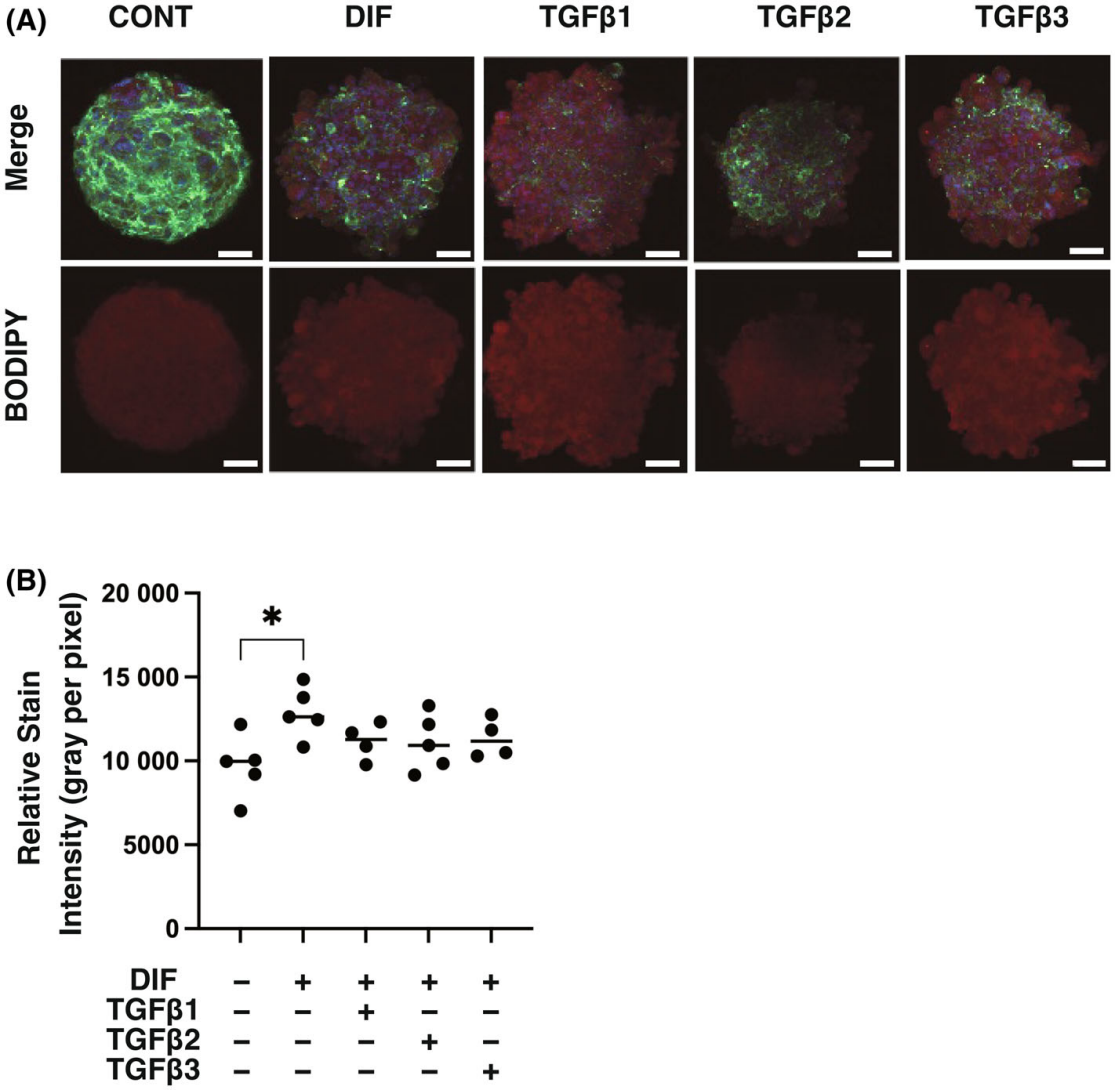
Effects of TGF‐β isoforms on mRNA expression of adipogenesis‐related genes of 3D 3T3‐L1 spheroids during their DIF^+^. 3D spheroids of 3T3‐L1 preadipocytes with adipogenesis (DIF^−^) or their adipogenesis (DIF^+^) with or without treatment with 0.1 nm TGF‐β1, TGF‐β2, or TGF‐β3 (*n* = 16 spheroids each) were immunostained with DAPI (blue), phalloidin (green) and BODIPY (red). Merge images and BODIPY images (each scale bar: 100 μm, panel A) and plots of their staining intensities (gray/pixel, panel B) are shown. *P < 0.05, **P < 0.01, ***P < 0.005, ****P < 0.001. The analysis is done by ANOVA with SD shown as error bars.

**Fig. 7:**
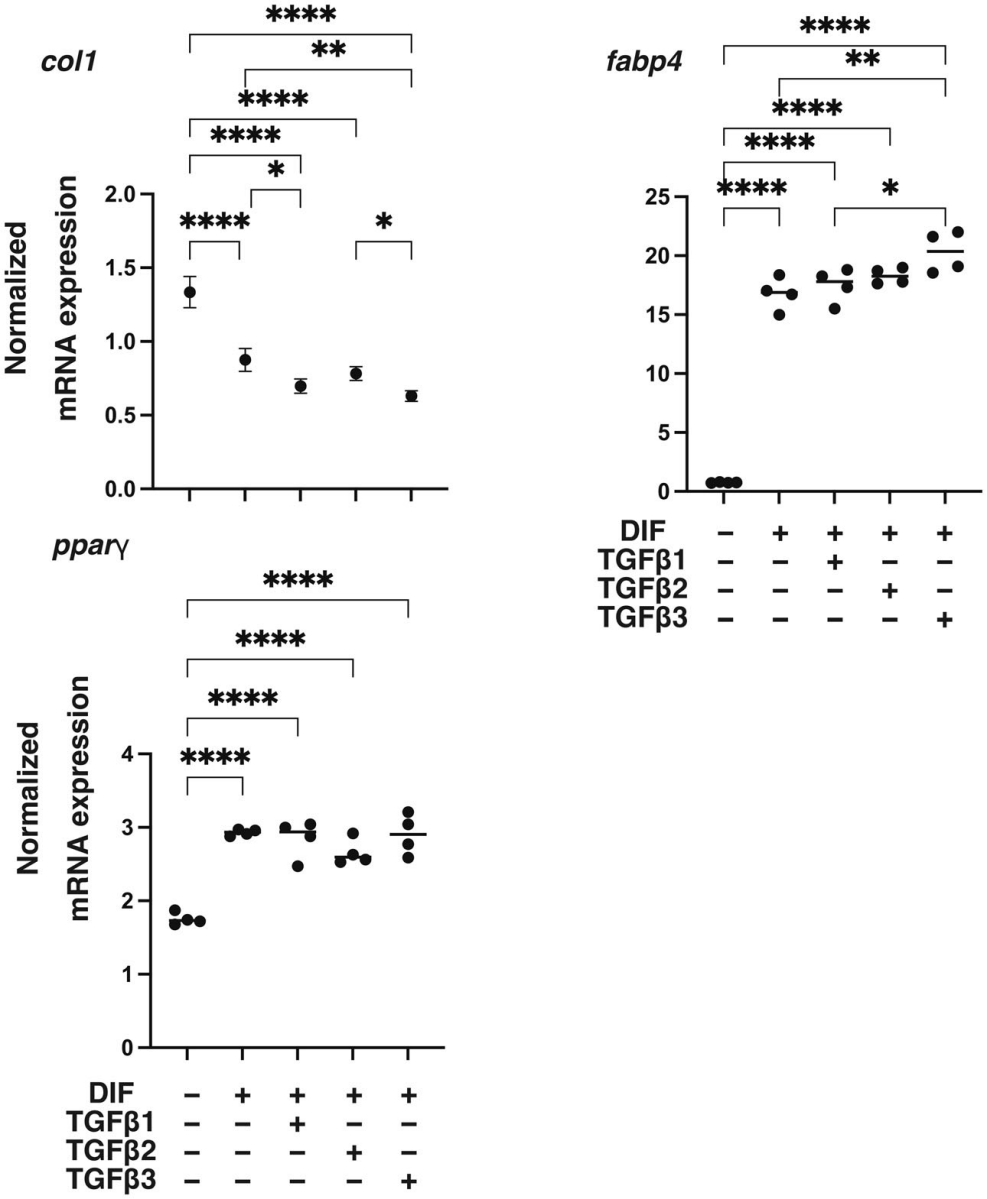
Effects of TGF‐β isoforms on mRNA expression of adipogenesis‐related genes, *pparγ*, and *fabp4*, and *col1*, of 3D 3T3‐L1 spheroids during their adipogenesis. 3D spheroids of 3T3‐L1 preadipocytes (DIF^−^) or their adipogenesis (DIF^+^) with or without treatment with 0.1 nm TGF‐β1, TGF‐β2, or TGF‐β3 (*n* = 16 spheroids each) were subjected to qPCR analysis of adipogenesis‐related genes including *pparγ*, *fabp4* and *col1*. All of the experiments were performed in duplicate using fresh preparations, each consisting of 16 spheroids. *P < 0.05, **P < 0.01, ***P < 0.005, ****P < 0.001. The analysis is done by ANOVA with SD shown as error bars.

Since the efficacy of adipogenesis of 3T3‐L1 cells was significantly different in 2D and 3D culture conditions [50, 51], the effects of the three TGF‐β isoforms on 3D 3T3‐L1 spheroids were also examined using their 3D spheroid cultures by following analyses: (a) measurements of mechanical aspects of the spheroids including size and stiffness, (b) lipid staining by BODIPY and (c) qPCR analysis for the above‐stated adipogenesis‐related genes. As shown in Fig. 5, the three TGF‐β isoforms had no significant effects on the size and stiffness of 3D 3T3‐L1 spheroids. Similarly, as shown in Fig. 6, the levels of lipid staining by BODIPY were increased by adipogenesis, but the increases in the levels of lipid staining were only slightly inhibited by the TGF‐β isoforms. In support of these results, qPCR analysis showed that the adipogenesis‐induced up‐regulation of *pparγ* and *fabp4* and down‐regulation of *col1* expression was not significantly changed by the TGF‐β isoforms (Fig. 7). Collectively, the results indicate that TGF‐β‐1~‐3‐induced suppression of adipogenesis observed in the 2D planar cell culture of 3T3‐L1 cells may be substantially minimized under the condition of 3D spheroid cell culture.

## Discussion

Although the exact role of TGF‐β during adipogenesis has remained unclear, it has been suggested that some members of the TGF‐β superfamily are crucial for MSC lineage decisions and adipogenic competency of committed preadipocyte cell lines [52]. In fact, TGF‐β expression has been revealed to positively correlate with obesity in humans and animal models [52, 53]. However, paradoxically, inhibition of adipogenesis by TGF‐β–SMAD3 signaling was also demonstrated in *in vitro* studies on adipogenesis of 3T3‐F442A cells [54] and 3T3‐L1 cells [40]. Although the reason for the discrepancy between the results of *in vitro* and *in vivo* studies has not been elucidated, the discrepancy may be due to differences in the ectopic activation of TGFβ–SMAD3 signaling in *in vitro* experimental conditions and *in vivo* states. However, as of this writing, although each of the three TGF‐β isoforms, TGF‐β‐1~‐3 has been suggested to be importantly involved in adipogenesis mechanisms, the biological roles of these three TGF‐β isoforms have not been compared in detail. In the current study, to elucidate this unidentified issue, we used 2D and 3D cultured 3T3‐L1 cells and observed different effects of TGF‐β isoforms on efficacy of adipogenesis of 3T3‐L1 cells analyzed by mRNA expression of adipogenesis‐related factors and lipid staining by Oil Red O or BODIPY and cellular metabolic functions related to mitochondria and glycolysis. Different effects of the three TGF‐β isoforms were also observed in both 2D and 3D cell culture conditions, but the diversity of the effects was much less in the 3D cell culture conditions than in the 2D cell culture conditions.

Much interest has been shown in the application of *in vitro* 3D spheroid models for a better understanding of physiological conditions as well as pathological conditions in various biological research fields [55]. In fact, compared with conventional 2D planar cultures, 3D spheroid cultures can provide biological natures that are close to those in *in vivo* states. For example, in 3D spheroid culture, cell‐to‐cell interaction can be observed at any locations unlike in conventional 2D cell culture, in which only side‐by‐side interaction can be observed. Therefore, such more flexible intercellular interactions of 3D spheroids can provide closer physiological environments in terms of protein networks such as several ECM proteins, cell junction proteins and others leading to suitable *in vitro* models replicating the biological natures of real tissues and organs [56]. Recently, we have used hanging drop 3D spheroid culture with a 384‐hanging drop array plate [57, 58] and we have succeeded in the formation of 3D spheroids originating from 3T3‐L1 preadipocytes [36]. Interestingly, we found that the 3D 3T3‐L1 spheroids we prepared had biological activities that were quite different from those of 2D cultured 3T3‐L‐1 cells despite identical experimental conditions except for the culture plates used for. That is, in contrast to 2D 3T3‐L1 cells, the 3D 3T3‐L1 spheroids induced spontaneous adipogenic differentiation without adipogenesis induction [50]. In support of this difference between 2D and 3D cultures of 3T3 L1 cells, in our recent study using RNA sequencing analysis, a total of 255 up‐regulated and 152 down‐regulated differentially expressed genes (DEGs) and a total of 124 up‐regulated and 58 down‐regulated DEGs were detected 2D and 3D cultured 3T3‐L1 cells, respectively, upon adipogenesis [51]. Gene ontology (GO) enrichment and ingenuity pathway analysis (IPA) analyses indicated that identified DEGs in the 3D spheroids were related to lipid metabolism among the top 4 causal networks related to disease and function (network score of more than 25) despite the fact that no network related to lipid metabolism was identified in the 2D cultured cells. Furthermore, seahorse metabolic analysis suggested that adipogenesis in 3D culture was more inducible for metabolic aspects of well‐differentiated adipocytes, and mRNA expression levels of mitochondria‐encoded genes were markedly increased by adipogenesis of 3D cultured 3T3‐L1 cells compared with adipogenesis of 2D cultured cells [51]. Since the significant effects of TGF‐β observed in the 2D cultured 3T3‐L1 cells may be masked during the more active adipogenesis‐related biological activities in 3D 3T3‐L1 spheroids, it is reasonable to speculate that the effects of TGF‐β on adipogenesis of 3T3‐L1 cells may be insignificant.

We acknowledge that there are some limitations in the present study. Firstly, only one cell line, preadipocyte cell line, 3T3‐L1, was used in this work, although this is the most extensively studied cell line in this adipocyte‐related research field. Therefore, to get more insight into the physiological significance of the effects of TGF‐β isoforms on adipogenesis, especially the adipocyte commitment of MSCs, additional studies using primary fat precursor cells, such as bone marrow stromal cell adipocytes [59], will be required. Secondly, since it has been have shown that TGF‐β/SMAD signaling has a critical role in the regulation of adipocyte commitment of MSCs and thus this mechanism could offer a new strategy for treating obesity, diabetes mellitus, and obesity‐related metabolism syndrome [54], contributions of various down‐stream factors of TGF‐β/SMAD signaling also need to be investigated. Thirdly, at present, we do not know the precise underlying mechanism of the diverse effects of the three TGF‐β isoforms on adipogenesis in 2D and 3D cultures of 3T3‐L1 cells. In our recent study, we found that six regulators including signal transducer and activator of transcription 3 (STAT3), interleukin 6 (IL6), angiotensinogen (AGT), FOS, and MYC, in addition to TGF‐β1, were possible master regulators for the formulation of a 3D spheroid architecture [50]. More interestingly, it has been shown that metformin stimulated anti‐adipogenic activities by regulation of the STAT3 and TGF‐β signaling cascades [60] and that JAK2/STAT3 signaling pathway plays an important role in lipid synthesis by which C/EBP β likes with STAT3 in preadipocytes [61]. Therefore, to obtain a better understanding of the physiological roles of TGF‐β isoforms in adipogenesis, we will investigate those scientific research subjects.

## Conflict of interest

The authors declare no conflict of interest.

### Peer review

The peer review history for this article is available at https://www.webofscience.com/api/gateway/wos/peer‐review/10.1002/2211‐5463.13890.

## Author contributions

AU and MW performed the experiments, analyzed data, and wrote the draft of paper. TS performed the experiments, analyzed data, and wrote the paper. MH and NN performed experiments and analyzed data. MF performed experiments, analyzed data and provided conceptual advice. HO designed experiments and wrote the paper. All authors have read and agreed to the published version of the manuscript.

## Supporting information


**Table S1.** Sequences of primers and Taqman and SYBR probes used are shown.

## Data Availability

The data that support the findings of this study are available from the corresponding author upon reasonable request.
