# Supplementary material for: TGF‐β effects on adipogenesis of 3T3‐L1 cells differ in 2D and 3D cell culture conditions
Source: FEBS Open Bio. 2024 Oct 8;14(12):2026–37. doi: 10.1002/2211-5463.13890 (PMC11609592; doi:10.1002/2211-5463.13890)
Supplement: Supplementary file 1 — Table S1. Sequences of primers and Taqman and SYBR probes used are shown. [file FEB4-14-2026-s001.pdf]

Taqman

Gene	Primer 1 (5' to 3')	Primer 2 (5' to 3')	Probe (5' to 3')	RefSeqNumber	Exon Location
rplp0 (36b4)	CGCTTGTACCCATTGATGATG	TTATAACCCTGAAGTGCTCGAC	/56-FAM/AGGCCCTGC/ZEN/ACTCTCGCTT/3IABkFQ/	NM_007475	5-6
adipoq	GCAGGATTAAGAGGAACAGGAG	TGTCTGTACGATTGTCAGTGG	/56-FAM/ACGACACCA/ZEN/AAAGGGCTCAGGAT/3IABkFQ/	NM_009605	1-2
cebpa	TCATTGTCACTGGTCAACTCC	ACAAGAACAGCAACGAGTACC	/56-FAM/CGCAAGAGC/ZEN/CGAGATAAAGCCAAAC/3IABkFQ/	NM_007678	1-1
fabp4	CCTTTCATAACACATTCCACCAC	AAATCACCGCAGACGACAG	/56-FAM/TGAAGAGCA/ZEN/TCATAACCCTAGATGGCG/3IABkF[illegible]	NM_024406	2-4
pparg	CTGCTCCACACTATGAAGACAT	TGCAGGTTCTACTTTGATCGC	/56-FAM/AGCTGACCC/ZEN/AATGGTTGCTGATTACA/3IABkFQ/	NM_011146	4-5

SYBR

Gene	Forward primer (5' to 3')	Reverse Primer (5' to 3')	RefSeqNumber
tgfb1r	TCTGCATTGCACTTATGCTGA	AAAGGGCGATCTAGTGATGGA	NM_009370
tgfb2r	GACTGTCCACTTGCGACAAC	GGCAAACCGTCTCCAGAGTAA	NM_009371
tgfb3r	GGTGTGAACTGTCACCGATCA	GTTTAGGATGTGAACCTCCCTTG☒	NM_0111578
